# Associations between low back pain, urinary incontinence, and abdominal muscle recruitment as assessed via ultrasonography in the elderly

**DOI:** 10.1590/bjpt-rbf.2014.0073

**Published:** 2015

**Authors:** Vânia F. Figueiredo, Juleimar S. C. Amorim, Aline M. Pereira, Paulo H. Ferreira, Leani S. M. Pereira

**Affiliations:** 1 Centro de Ciências Biológicas e Saúde, Pontifícia Universidade Católica de Minas Gerais (PUC Minas), Belo Horizonte, MG, Brazil; 2 Departamento de Saúde Pública, Universidade Estadual de Londrina (UEL), Londrina, PR, Brazil; 3 Prefeitura de Belo Horizonte (NASF), Belo Horizonte, MG, Brazil; 4 Faculty of Health Sciences, University of Sydney, Sydney, NSW, Australia; 5 Departamento de Fisioterapia, Universidade Federal de Minas Gerais (UFMG), Belo Horizonte, MG, Brazil

**Keywords:** elderly, low back pain, urinary incontinence, physical therapy, ultrasound imaging

## Abstract

**Background:**

Low back pain (LBP) and urinary incontinence (UI) are highly prevalent among elderly individuals. In young adults, changes in trunk muscle recruitment, as assessed via ultrasound imaging, may be associated with lumbar spine stability.

**Objective:**

To assess the associations between LBP, UI, and the pattern of transversus abdominis (TrA), internal (IO), and external oblique (EO) muscle recruitment in the elderly as evaluated by ultrasound imaging.

**Method:**

Fifty-four elderly individuals (mean age: 72±5.2 years) who complained of LBP and/or UI as assessed by the McGill Pain Questionnaire, Incontinence Questionnaire-Short Form, and ultrasound imaging were included in the study. The statistical analysis comprised a multiple linear regression model, and a *p*-value <0.05 was considered significant.

**Results:**

The regression models for the TrA, IO, and EO muscle thickness levels explained 2.0% (R^2^=0.02; *F*=0.47; *p*=0.628), 10.6% (R^2^=0.106; *F*=3.03; *p*=0.057), and 10.1% (R^2^=0.101; *F*=2.70; *p*=0.077) of the variability, respectively. None of the regression models developed for the abdominal muscles exhibited statistical significance. A significant and negative association (*p*=0.018; β=-0.0343) was observed only between UI and IO recruitment.

**Conclusion:**

These results suggest that age-related factors may have interfered with the findings of the study, thus emphasizing the need to perform ultrasound imaging-based studies to measure abdominal muscle recruitment in the elderly.

## Introduction

Population aging is occurring worldwide. In Brazil, given the demographic and epidemiological evolution of chronic diseases, population aging requires constant care and monitoring and thus increases the demand for health services^1^.

Low back pain (LBP)^2^ and urinary incontinence (UI)^3^ are conditions that strongly affect functioning in the elderly and hinder the performance of everyday activities, thus causing physical and emotional distress, incurring high socioeconomic costs, restricting social participation, and decreasing the quality of life^4^. Moreover, LBP and UI are erroneously considered natural aspects of the aging process^3^. Approximately 50-80% of the general population appears to have experienced at least 1 episode of LBP during their lifetime^1^. The prevalence of LBP remains stable and ranges from 12-33%^5^ worldwide, with rates of approximately 63% in the Brazilian population and 57.7% among elderly individuals^1^. The annual incidence of UI in women ranges from 2-11%, and this disorder is twice as common in women as it is in men^6^.

In healthy individuals, the abdominal and pelvic floor muscles work synergistically^7^^-^ 10. However, in the absence of micturition control, the pelvic muscle activation pattern apparently changes and overloads the spine stabilizers^3^^,^ 10. The pelvic floor muscles play an important role in the provision of postural lumbo-pelvic stability, which is conferred by connections of the muscles around the trunk^7^^,^ 8 ^,^ 10.

Because of its anatomic characteristics, the transversus abdominis (TrA) muscle preferentially stabilizes the spine^11^^,^ 12. Moreover, the TrA is the first muscle to be activated in response to lower and upper limb movements, thus conferring the required rigidity to the lumbar spine and avoiding undesired segmental movements^13^. Delayed TrA activation is observed in younger adults with chronic LBP and suggests a failure in lumbo-pelvic stabilization^12^^-^ 14.

In the elderly, stabilization failure due to geometric muscle and postural alterations can occur because the musculoskeletal and nervous systems are influenced by a variety of pathophysiological changes that lead to uncoordinated performance^15^^-^ 17, including decreased maximal voluntary contractions^15^; reductions in the peak muscle power^15^, transverse area, and rate of neuromuscular activation; increased intramuscular fat deposits^17^; and reductions in the muscle fiber length (atrophy) and number (hypoplasia), which particularly affects hybrid fibers^15^. Changes might also occur in sensory receptors, peripheral nerves, joints, and the central nervous system (e.g., decreases in white and gray matter volume and dopaminergic denervation)^18^. This complex array of modifications responsible for age-related losses of muscle mass is collectively called sarcopenia and occurs due to hormonal, nutritional, immunological, and metabolic alterations^15^. Sarcopenia can be triggered by changes in either the intracellular signaling cascade or the basic cellular processes that inhibit satellite cell activation, particularly during inflammation^19^.

Ultrasound imaging (i.e., rehabilitative ultrasound imaging [RUSI]) has been accepted as a valid tool for assessing muscle recruitment because similar results can be obtained via electromyography (EMG) evaluation^12^^,^ 20 ^,^ 21. The main advantage of ultrasound imaging is its low invasiveness^12^^,^ 20 ^,^ 21. Physical therapists use ultrasound measurements to assess muscle function and soft tissue morphology during movement or while performing specific tasks^20^^,^ 22. Ultrasound imaging is also used to assist therapeutic approaches intended to improve neuromuscular function^14^^,^ 20 ^,^ 23 ^,^ 24.

Several studies have described the neuromuscular trunk muscle patterns via ultrasound imaging in young adults both with and without a history of low back pain^12^^-^ 14 ^,^ 21. However, the relevance of these findings in elderly populations is unknown, as no studies involving ultrasound imaging in elderly individuals with LBP were found in the reviewed literature^2^.

Accordingly, the present study analyzed the association between LBP and UI as well as the patterns of TrA, internal oblique (IO), and external oblique (EO) muscle recruitment as determined via ultrasound imaging in a cohort of community-dwelling elderly individuals.

## Method

### Individuals

Data were collected from male and female community-dwelling elderly individuals who were aged 65 years or older and had no cognitive alterations as assessed using the Mini Mental State Examination (MMSE)^25^. These individuals had complained of LBP, and some had reported UI. The exclusion criteria were acute low back pain; evidence of radiculopathies (e.g., reflex alteration, dermatomes, and/or myotomes or positive Lasegue test); a positive clinical history of neurological diseases, thoraco-abdominal surgeries (e.g., cesarean delivery and hysterectomy) or spinal surgeries, and vertebral fractures; signs suggestive of severe spinal cord injury due to severe trauma; a history of malignant tumor (prostate cancer) or unexplained weight loss; severe spinal deformities (e.g., scoliosis, hyperkyphosis); spinal physical therapy in the last 6 months; and/or lumbar and/or pelvic floor stabilization exercises.

The sample size was calculated while considering the abdominal muscle recruitment pattern as well as the dependent and independent study variables, the presence of LBP, and reported UI. It was calculated that 54 subjects would be required to obtain a correlation of 0.40 between the dependent and independent variables and a correlation of 0.20 among the independent variables with a coefficient of determination (R^2^) of 0.40 and a statistical power of 80%. For convenience, we sequentially recruited 60 community-dwelling elderly individuals. After selection and initiating data collection, 6 participants were excluded from the study. Therefore, the statistical analyses included 54 participants.

### Study design

This cross-sectional observational study was approved by the Research Ethics Committee of the Universidade Federal de Minas Gerais (UFMG), Belo Horizonte, MG, Brazil (ETIC 324/07) and was conducted according to Resolution 196/96 of the National Health Council, which addresses the Code of Ethics of Human Research. After having read and obtained clarification regarding the study terms, each individual signed an informed consent form prior to participation.

### Materials and procedures

The cohort was assessed using a questionnaire that included questions about sociodemographic and clinical-functional information. LBP was characterized using the McGill Pain Questionnaire (Br-MPQ)^4^^,^ 26, an appropriate tool for assessing chronic pain in elderly individuals. The intra- and inter-examiner reliability rates for the Brazilian version of the Br-MPQ were found to be 0.86 and 0.89, respectively, with rates of 0.71 and 0.68 for orthopedic and neurological diseases, respectively^4^.

To assess the presence of UI and determine the frequencies and amounts of urinary loss reported by the participants, 2 questions from the International Consultation on Incontinence Questionnaire-Short Form (ICIQ-SF) quality of life survey that were UI-specific and had been validated for Portuguese-speaking subjects were implemented^27^.

**Figure 1:**
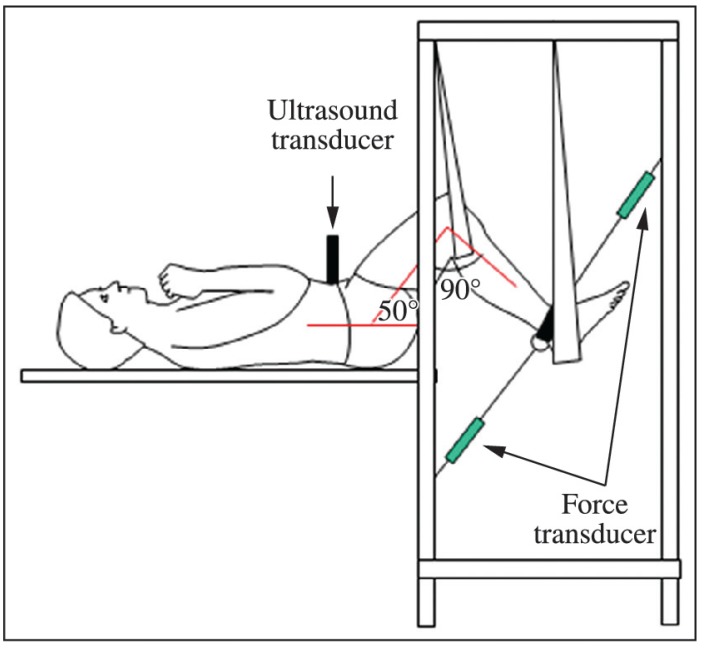
Diagram of the device used to position the participant during ultrasound image collection. Source: Ferreira et al.12 (used with permission).

A 2-dimensional ultrasound imaging device (Sonoline SL1; Siemens Healthcare, Erlangen, Germany) was used to evaluate abdominal muscle recruitment. The images were captured by a 10-cm, 7.5-MHz transducer coupled to the ultrasound imaging device. A more detailed description of the protocol used for our assessments and measurements was provided in the original study^21^. To ensure intra- and inter-examiner reliability, a pilot study with 12 volunteers was conducted. The test-retest reliability results obtained using the intraclass correlation coefficient (ICC) were 0.76 (95% CI: 0.16-0.93) for the TrA, 0.49 (95% CI: -0.76-0.85) for the IO, and 0.58 (95% CI: -0.46-0.88) for the EO. All ultrasound images were captured and analyzed by the same previously trained researcher. Each participant was asked to lie on a stretcher, and the researchers positioned the lower limbs using a device with a rectangular metal frame according to a previous model^12^^,^ 21. The limbs were positioned to allow the hips and knees to remain flexed at 50º and 90º, respectively (Figure 1). The participant was then asked to cross their arms over their chest, and the ultrasound transducer was positioned at the height of the umbilical scar, which was approximately 10 cm from the midline, lateral to the abdominal wall, and between the iliac crest and rib cage. After proper positioning, the participant was asked to remain at rest while images of the abdominal muscles at rest (baseline) were captured. Next, the participant was instructed to generate a contraction force before bending and then extending the knees; this corresponded to 7.5% of the body mass. This force produced an isometric contraction of the abdominal muscles, which was measured using a force-gauge (Cabela's^(r)^ Digital Scale; Cabela's Incorporated, Sidney, NE, USA). The images were stored using video software (Pinnacle Studio, version 9.4^(r)^; Corel Corporation, Ottawa, ON, Canada).

### Statistical analysis

A descriptive analysis of the quantitative variables was conducted by calculating the average, a central trend measure, and the standard deviation that assessed the sample data variability. A descriptive analysis of the qualitative variables was conducted by calculating the frequencies of each category.

To evaluate the associations between the continuous variables, 3 multiple linear regression analysis models were created. Each model used the proportion of the abdominal muscle (TrA, IO, and EO) recruitment as the dependent variable and the LBP and UI as independent variables.

All statistical analyses were performed using the Statistical Package for the Social Sciences (SPSS) for Windows (version 17.0; SPSS Inc., Chicago, IL, USA). The level of significance was set at *p*<0.05.

## Results

The sociodemographic and clinical characteristics of the study cohort are presented in Table 1. The cohort mostly comprised women (76%) and after calculating the body mass index (BMI) values of the participants, 46.3% of the subjects were determined to be eutrophic according to the interval (22-27 kg/m^2^) for elderly individuals as proposed by Lipschitz^28^; an additional 14.8% were malnourished, and 38.9% were overweight. Among the elderly patients suffering from LBP (n=34), 52.9% reported moderate pain (2.24±0.78) and 44.1% reported short/transitional/temporary pain according to the Br-MPQ survey (Table 1). Among the elderly patients reporting UI (n=22), 54.5% were losing urine once weekly or less frequently, and 81.8% ranked this loss as of low intensity.

**Table 1:** Sociodemographic and clinical characteristics of the studied cohort (n=54).

Sociodemographic and clinical characteristics	n (%)	Mean±SD	Range
Age (years)	54 (100%)	72±5.2	65-84
Gender (Female)	41 (76%)		
School education (year)			
Analphabet	5 (9.3%)		
1-8	39 (72.2%)		
≥9	11 (20.3%)		
Number of comorbidities	-	4.78±2.35	1-13
Number of drugs			
None	7 (13%)		
1-5	38 (70.3%)		
>5	9 (16.7%)		
UI *(yes)*	22 (40.7%)		
LBP (*yes)*	34 (62.9%)		
Pain intensity (Br-MPQ)^a^		1.41±1.25	0-4
Mild (1)	5 (14.7%)		
Moderate (2)	18 (52.9%)		
Severe (3)	9 (26.5%)		
Unbearable (4)	2 (5.9%)		
Pain temporal pattern (Br-MPQ)^a^			
Continuous/stable/constant	10 (29.4%)		
Rhythmic/periodic/intermittent	9 (26.4%)		
Brief/momentary/transitory	15 (44.1%)		

SD: standard deviation; %: relative percentage of elderly individuals (n=54); Br-MPQ: McGill pain questionnaire-Brazil; UI: urinary incontinence; LBP: low back pain;

a Values for n=34 elderly patients who reported the presence of LBP.

None of the clinical variables strongly correlated with the TrA (UI, *p*=0.541; LBP, *p*=0.412) and EO muscle thicknesses (UI, *p*=0.091; LBP, *p*=0.078). Furthermore, LBP was not associated with the IO muscle thickness (*p*=0.931). Table 2 shows the results of the linear multiple regression models used to assess correlations between the TrA, IO, and EO muscle recruitment patterns and the variables of LBP and UI. These results illustrated that the regression models for the TrA, IO, and EO muscle recruitment levels explained 2.0% (R^2^=0.02; *F*=0.47; *p*=0.628), 10.6% (R^2^=0.106; *F*=3.03; *p*=0.057), and 10.1% (R^2^=0.101; *F*=2.70; *p*=0.077) of the variability, respectively. Only the model for the IO muscle was statistically significant.

**Table 2:** Associations between the TrA, IO, and EO recruitment levels and LBP and UI.

Variables	TrA		IO		EO	
	Β	p	b	p	Β	p
Constant (b_0_)	0.039	0.015^a^	0.0303	0.015^‡^	0.0001	0.992
UI (b_1_)	–0.011	0.541	–0.0343	0.018^‡^	0.0218	0.091
LBP (b_2_)	0.015	0.412	0.0012	0.931	–0.0232	0.078

UI: urinary incontinence; LBP: low back pain; Multiple linear regression analysis;

a p<0.05;

‡ p<0.05 Stepwise multiple linear regression.

## Discussion

This study analyzed the associations between LBP and UI and abdominal muscle (TrA, IO, and EO) recruitment patterns as measured by ultrasound imaging in a cohort of community-dwelling elderly individuals. According to our findings, no abdominal muscle recruitment could be used to explain the LBP and UI variables. However, UI was significantly negatively associated (*p*=0.018; β=-0.0343) with IO recruitment; in other words, elderly individuals who exhibited higher IO activation reported lower urine losses. This finding partially confirmed those of previous studies in which the IO and TrA were reportedly predominantly recruited during pelvic floor muscle contraction, thus controlling continence through bladder stabilization and increased intra-urethral pressure^7^^,^ 8.

The co-activation of the abdominal muscles and pelvic floor is consistent with the model in which the muscles surrounding the abdominal cavity were predicted to work together in a coordinated manner to ensure column stability and maintain continence^7^^,^ 8.

In a recent review study conducted by Ferreira and Santos^29^, the synergistic activation of the deep abdominal muscles (i.e., the lower fibers of the TrA and IO) was described during pelvic floor muscle contraction such that TrA contraction led to pelvic floor muscle co-contraction. These authors also suggested that abdominal muscle contraction should not be discouraged during pelvic floor muscle exercises because this would limit the pubococcygeus muscle response without producing a significant increase in intra-abdominal pressure.

One possible explanation of this finding relies on the characteristics of the studied sample, which comprised elderly individuals with an average age of 72±5.2 years and complaints of LBP and/or UI. Among the individuals complaining of UI (n=22), 72.7% (n=16) also complained of LBP, a factor that may have influenced the observed results. The previous studies^7^^,^ 8 were conducted in a cohort of young adults with no history of LBP and/or UI. Therefore, we suggest that the association observed between UI and IO recruitment in this study be interpreted with caution and that further studies intended to classify these individuals be performed.

The lack of association between EO recruitment and the variables of LBP and UI concurred with previously reported results^12^ and indicates that ultrasound imaging is likely not a valid instrument with which to measure EO recruitment, given the poor correlation between the EMG results and ultrasound images observed for this muscle (R=0.28).

TrA muscle recruitment variability was not associated with either LBP or UI in the present study sample. The trunk muscle recruitment pattern observed via ultrasound imaging in young adults with and without LBP has been highlighted in the literature^12^^-^ 14 ^,^ 20 ^,^ 21. During muscle contraction, although EMG detects the production of action potentials, changes in the muscle shape and geometry are also noted. These changes enable the ultrasound imaging-based measurement and recording of changes in the muscle thickness during contraction^11^^,^ 12 ^,^ 21.

The elderly exhibit changes in movement and motor control^15^^-^ 17. The former are due to sarcopenia, osteopenia, reduced sensory and motor proprioception, postural and biomechanical compensatory changes, and reduced nerve conduction velocity^15^. Losses of muscle mass, losses and atrophy of muscle fibers (particularly more marked losses of type II fibers [i.e., fast glycolytic contraction fibers])^16^, and losses in the size and number of motor units^30^ are responsible for the decreases in muscular strength, power, and endurance^15^^,^ 30 observed during aging.

The protocol used in this study^21^ was developed and tested in young adults. It is possible that the low force generated by the isometric muscle contraction used in this study (7.5% of the body mass) did not allow the detection of changes in the TrA thickness, given that typical age-related alterations can affect the structure and composition of muscles.

Singh et al.^16^ compared changes in the lumbar extensor muscles, fiber orientations, and muscle strength in young and elderly subjects using ultrasound imaging and found that age-related changes interfered with both muscle geometry and posture. Moreover, changes in the spinal curvature and consequently the body position and joint movements might affect the muscle contraction and lever strength^13^^,^ 14. The loss of lumbar lordosis might affect the muscle length, fiber orientation, and fascicle geometry, thus affecting muscle strength^16^^,^ 30.

In summary, the lack of existing studies in the literature that incorporate ultrasound imaging to determine the abdominal muscle recruitment pattern in elderly individuals with complaints of LBP and UI made it difficult to compare the results observed in this study with those reported in the literature.

Regarding the association between LBP and UI, this study pioneered the evaluation of the TrA, IO, and EO muscle recruitment pattern via ultrasound imaging in elderly individuals. The multiple linear regression models used to verify this association revealed that only UI exhibited a significant association with IO recruitment. These results differed from those observed in young adults. Inherent age-related factors such as sarcopenia, changes in motor control, and the ultrasonography technique all possibly interfered with the findings of this study.

As age-related changes affect the entire body, further investigations involving ultrasound imaging will be required to identify the effects of aging on the recruitment patterns of these muscles.
